# Adapting the one minute preceptor model to a Swedish context – clinical tutors’ experiences of its usage in primary health care

**DOI:** 10.1080/02813432.2025.2508370

**Published:** 2025-05-26

**Authors:** Erica Rothlind, Helena Salminen, Katarina Rolfhamre, Klas Karlgren, Eva Toth-Pal

**Affiliations:** ^a^ Academic Primary Healthcare Centre, Region Stockholm, Sweden; ^b^ Department of Neurobiology, Care Sciences and Society, Karolinska Institutet, Stockholm, Sweden; ^c^ Unit for Teaching and Learning, Karolinska Institutet, Stockholm, Sweden; ^d^ Stockholm Health Care Services, Region Stockholm, Sweden; ^e^ Department of Learning, Informatics, Management and Ethics (LIME), Karolinska Institutet, Stockholm, Sweden; ^f^ Department of Research, Education, Development and Innovation, Education Center, Södersjukhuset, Stockholm, Sweden; ^g^ Faculty of Health and Social Sciences, Department of Health and Functioning, Western Norway University of Applied Sciences, Bergen, Norway

**Keywords:** One minute preceptor model, clinical tutor, clinical supervision, undergraduate students, clinical reasoning, feedback, primary health care

## Abstract

**Background:**

The One Minute Preceptor model is one of few models proven to be effective in clinical training and validated for teaching clinical reasoning and medical knowledge. It has been introduced to tutors from various health care professions and is considered easy to learn. It is, however, not well-known in Scandinavian primary health care, and studies introducing the model in this context are lacking. Moreover, qualitative studies exploring the views of tutors using the model are also lacking. The aim was therefore to adapt the One Minute Preceptor model to a Swedish context and to explore tutors’ experiences of applying it in a primary care setting. A qualitative method with quantitative elements was used.

**Methods:**

A workshop was designed and held in interprofessional groups at various primary health care centres in Region Stockholm. Follow-up interviews were conducted. Data were analysed using qualitative content analysis. Furthermore, pre- and post-surveys were administered.

**Results:**

The results showed that the tutors believed the model to be flexible, and easy to use and adapt, and their self-rated competence increased. Moreover, they believed it could help them give feedback and explore the students’ reasoning. This is in line with previous research, but our study contributes by adding the tutor’s *how* perspective on how the model affects these key elements of clinical tutoring.

**Conclusions:**

In conclusion, interprofessional workshops teaching the One Minute Preceptor model are readily held and may be feasible to scale up. Positive outcomes on clinical tutoring are likely; especially on feedback and clinical reasoning, where improvements are needed.

## Background

Medical and nursing students spend much of their training at primary health care (PHC) centres, where a unique opportunity to link theory with practice is offered. Here, guidance from clinical tutors is essential [1].

Literature shows that effective clinical tutoring requires clinical competence, interpersonal skills, and teaching and supervisory skills, which include the ability to provide constructive feedback [1,2]. Although clinical tutoring is complex, it is also considered personally rewarding; gaining new perspectives on work and learning from the student have been highlighted benefits [3–5]. Nonetheless, barriers to tutorship are highly prevalent according to the literature. For example, the role of the clinical tutor is often described as two-fold: the difficulty of combining patient care with tutoring has been identified as a main barrier [6]. Other barriers include upholding productivity quotas and managing disruption to workflow, lack of preparedness and formal training, and limited knowledge of teaching techniques [4,6–9].

Training tutors to use structured teaching strategies, that translate educational theory into practice may ease their burden and improve effectiveness [10]. One of the few well-established models that has consistently proven effective in clinical training is the One-Minute Preceptor model (OMP) [11,12]. The OMP aligns well with theories of experiential learning emphasised in contemporary medical education. In summary, experiential learning theories describe education as a process of individual transformation in reaction to experience and perception [13,14]. Moreover, the OMP has been highlighted by Savaria et al. as one of the few methods grounded in educational theory that has been validated for teaching clinical reasoning, medical knowledge and clinical skills [15].

The OMP was developed for clinical tutors in PHC, anddescribed as efficient for balancing patient care with clinical tutoring [16]. The model was designed with the intention of increasing the frequency and quality of tutoring sessions in clinical settings, where time constraints are a well-known limitation. The OMP is considered readily taught to tutors and possible to implement at a low cost as the only prerequisite for using the model is understanding the ‘micro-skills’ involved [12,17]. Previous studies have shown that a relatively brief training session suffices [18,19]. In the original model [16] the five micro-skills are described as follows: 1) Get a commitment; 2) Probe for supporting evidence; 3) Teach general rules; 4) Reinforce what was done right; and 5) Correct mistakes.

By using these micro-skills, students’ clinical reasoning and case presentation skills have been shown to improve [12]. Moreover, after OMP training, tutors report that they are more confident in several teaching skills, including providing specific feedback and allowing the student time to reason [19]. Both tutors and students preferred the OMP to traditional teaching methods [20].

The OMP was originally developed for post-graduate trainees in a PHC setting, but has since been applied in various student populations and hospital settings, including emergency and obstetrics departments [17,18,21,22]. Moreover, adaptations for nursing education have been published [23,24].

Most OMP studies are quantitative, often using tutor and learner surveys [25–27]. One study combined tutors’ self-evaluations with an analysis of recorded tutoring sessions and found that OMP increased the specificity of feedback [19]. In another mixed methods study, interviews with teachers in paediatrics residency training found that the OMP was well received and concluded that it can be used as a complement to traditional teaching methods [28]. Except for this hospital-based study, we have not found other qualitative studies exploring tutors’ experiences of OMP.

In addition, there is a need for studies on OMP training for both medical and nursing tutors. Given the existing literature on shared concerns in tutorship [4,6–9], we postulated that interprofessional training may be both feasible and useful. Finally, studies are lacking when it comes to introducing the OMP in Scandinavian PHC.

The aim of the study was therefore to adapt the OMP to a Swedish context and to explore tutors’ experiences of applying the model in a primary care setting.

## Methods

### Design

The study was a qualitative study with some quantitative elements, exploring the experiences of tutors following training and application of the OMP in clinical tutorship in PHC.

An exploratory qualitative research approach was chosen since the study focused on exploring the views and experiences of medical and nursing tutors. The study was guided by constructivist theories of learning, where knowledge is considered a product of interaction between individuals and society, which fit well with the use of qualitative methodology [29].

The project was conducted in four phases: 1) needs assessment interviews; 2) design of an OMP workshop; 3) holding the OMP workshops; 4) follow-up through interviews and a survey.

#### Needs assessment interviews

A needs assessment was conducted through three digital group interviews (*N* = 8; five specialists in family medicine and three nurses with experience of clinical tutorship). Incentives and barriers for tutorship in primary care and how they can be addressed through training were discussed. The OMP model was not introduced, since we wanted to gather broad perspectives without influencing responses. Concerns regarding time management and feedback were raised, underscoring the potential relevance of the OMP.

#### Designing an OMP workshop

The second phase involved designing an OMP workshop for clinical tutors in primary care, aimed at both physicians and nurses. It was structured as a 1.5-hour formal training session, and included a PowerPoint presentation addressing the key elements of OMP, an example video of an OMP session (scripted and recorded by the research team) and a role-play activity. Insights from the needs assessment interviews guided the design of the workshop. For example, since role plays were seen as valuable but acting in front of others was a concern, participants practiced in pairs without an audience.

The five micro-skills were translated into Swedish, and the wording was adapted to fit the present medical education context. ‘Correct mistakes’ was replaced with ‘feedforward’, as the latter is in line with more contemporary models for feedback [30]. The concept of feedforward refers to identifying specific actions students must take to improve their performance, and is a central part of the feedback process [31]. Likewise, ‘rule of thumb’ was chosen instead of ‘general rules’ to incorporate experience-based knowledge.

**Figure 1.**
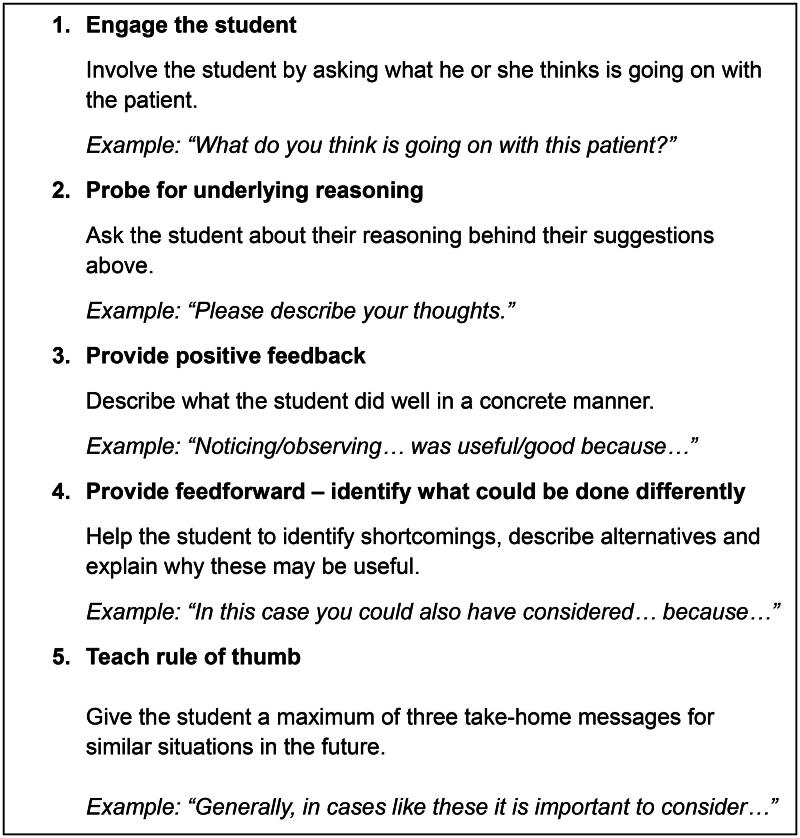
The five micro-skills of the OMP as presented in the workshop and outlined on the laminated cards. (translated and adapted by the research team.).

Laminated cards outlining the micro-skills, and example questions were provided (Figure 1).

On the laminated cards, ‘teach rule of thumb’ was placed last, though participants were told they could also use it prior to the feedback/feedforward. This order was chosen to reinforce feedforward and support learning, in line with recent OMP adaptations [15].

#### Holding the OMP workshops

The third phase involved holding the workshop at various PHC centres.

- *Setting and participants*
  In Region Stockholm, which was the setting for this study, there are both public and private PHC providers accredited by the region. Their responsibilities include facilitating clinical placement and tutoring for health care students. Medical and nursing students are the largest groups, with the placement duration varying between programmes and universities.
  The PHC centres were conveniently sampled to ensure a collective tutoring experience at the unit [32]. Inclusion criteria were ongoing tutorship for medical students (year three or higher), or nursing students (year two or higher), and having 8000 or more registered patients.
  The administration office (at Karolinska Institutet’s Division of Family Medicine and Primary Care) provided e-mail addresses for contact persons at eligible PHC centres. Invitation e-mails were sent to 62 PHC centres, offering an OMP workshop for clinical tutors on-site. The e-mail outlined the aim of the workshop and explained that it would be followed up by a group interview with the participants. The contact persons selected participants at their respective units. In total, 93 tutors at 11 PHC centres attended the workshops.
  Participants were asked to fill out a pre-survey at the start of the workshop (Appendix 1). It covered background information and 13 Likert-scale questions on their confidence in tutoring and use of various micro-skills (not named as such in the survey). In total, 87 participants submitted the pre-survey. Their characteristics are summarised in Table 1.
- *The OMP workshop*
  The workshop was conducted by the authors ER, ETP and KR at all sites. At the end of each workshop, time was allowed for questions and discussions. Participants were given access to the presentation, videos and additional OMP material via a website (https://ki.instructure.com/courses/17949) created by the research team. They were asked to share these only with colleagues at their own clinic. Laminated cards summarising the key elements of the OMP (as outlined above in Figure 1) were distributed.

**Table 1.** Characteristics of the 87 participants submitting the pre-OMP workshop survey, all of whom also completed the OMP workshop.

Profession	Physician (*N* = 37)	Nurse (*N* = 50)
Gender (F/M)	20/17	47/3
Age, median (range)	44 (31–65)	44 (23–69)
Years of clinical work, median (range)	10 (1–30)	15 (1–45)
Years of clinical tutoring, median (range)	7.5 (0*–18)	13 (0.5–43)
Number of weeks of tutoring/year, median (range)	2 (0*–15)	8 (3–16)

*One of the physicians was due to start tutoring after the workshop.

Finally, participants were asked to rate the included activities on a five-point Likert scale and provide feedback in an evaluation form.

#### Data collection through follow-up interviews and surveys

In the fourth phase, the intervention was evaluated after seven to 11 months through group interviews and surveys.

##### Interviews

**Figure 2.**
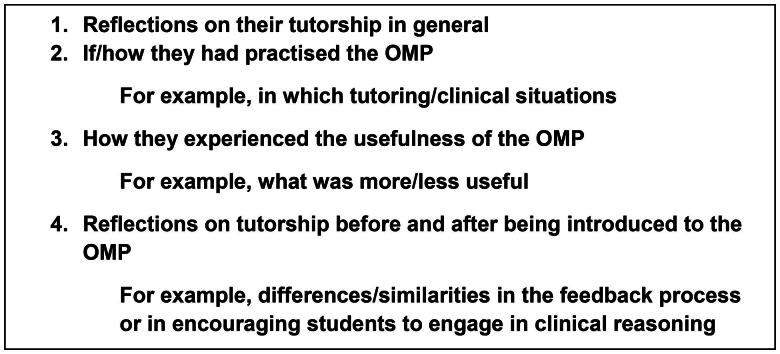
A summary of the topic guide for follow-up interviews with tutors on their experiences of using the OMP.

Qualitative data were collected through group interviews at each participating PHC centre. Due to logistical reasons, we were not able to mix participants from different centres. A topic guide with open-ended questions was constructed by the authors with the intention of prompting discussion on experiences of using the OMP. The same guide was used for physician and nursing tutors, as almost all interviews were held in mixed groups, allowing for interprofessional exchange. Follow-up questions were used to elicit more detailed information. The guide is summarised in Figure 2.

Each interview was conducted by two of the three authors ER, ETP and KR. ER and ETP moderated and observed crosswise and KR observed. There was a discussion within the research team on whether an external interviewer or a member of the team should conduct the interviews. We chose the latter, valuing familiarity with the workshop and the participants’ concerns, as the focus was on OMP application rather than evaluating the workshop as such.

Interviews were held in Swedish, lasted 32 -55 min (mean: 42), and were audio recorded, transcribed verbatim, and conducted until saturation. Field notes were taken by the observer.

##### Participants

In total, 26 tutors from eight of the 11 PHC centres participated in the follow-up interviews. These tutors were selected by the each centre’s contact person. The aim was to conduct focus group interviews; however, this was not feasible due to fewer informants than anticipated attending the interview at some of the centres. Altogether, eight group interviews were held, with two to five informants each. There were 20 women and six men, reflecting the gender distribution of those participating in the workshop. The distribution between physicians and nurses was equal.

##### Post-survey

The post-survey included questions corresponding to the pre-survey, with response options on a five-point Likert scale (Appendix 2). The third item, regarding the need for competence development was not repeated, sinceit was used as an indicator of the tutors’ estimated need. The post-survey was first distributed electronically *via* a survey client at Karolinska Institutet to all participants during the semester following their respective workshop. Two reminders were sent out. As an alternative option, the participants were also e-mailed the survey which they could choose to print and return anonymously by mail. However, as the response rate was low, participants in the follow-up interviews were asked to fill out the survey in paper form before the interview if they had not previously responded. Consequently, all interview participants also completed the post-survey; their characteristics are summarised in Table 2.

**Table 2.** Characteristics of the 32 participants submitting the post-OMP workshop survey; all but six also participated in the interviews.

Profession	Physician (*N* = 15)	Nurse (*N* = 17)
Gender (F/M)	10/6	16/1
Age, median (range)	46 (32–66)	49 (23–63)
Years of clinical work, median (range)	11 (3–32)	12 (2–37)
Years of clinical tutoring, median (range)	8 (0.5–20)	12.5 (0.5–26)
Number of weeks tutoring/year, median (range)	3 (1–10)	8 (2–14)

##### Student survey

Our ambition was also to compare student experiences of clinical tutorship at the OMP-trained units with those units who applied tutoring ‘as usual’. An electronic survey was sent over three semesters to 1107 nursing students (semesters 4–5) and 2062 medical students (semesters 5–11), asking them to identify their PHC centre and rate how often supervisors applied OMP micro-skills (described but not named as such). Despite reminders, response rates were very low (Table 3), especially from students at participating centres. Therefore, after discussion with statisticians, data were deemed not feasible for statistical analysis. In summary, the overall scores were high, means ranged from 3.2 to 4.2, on a scale from 1 to 5.

**Table 3.** The response rate for the survey administered to medical and nursing students on clinical rotation in primary care, with data split into an OMP intervention and a control group.

Student	Number of surveys sent out	Number of responses		Total	Response rate %
		Intervention	Control		
Nursing	1107	12	262	274	25
Medical	2062	38	227	265	13
Total	3169	50	488	538	17

#### Data analysis

##### Qualitative

Data were analysed using qualitative content analysis [33]. This was deemed a suitable method since the present study aimed to explore personal experiences [33]. In accordance with the method, the material was read repeatedly to gain an overview.

The transcripts were systematically coded line by line, with units of meaning extracted to an Excel sheet. They were condensed while preserving core content and then assigned manifest codes. Codes addressing the same issue were grouped into descriptively labelled sub-categories, ensuring inclusion of both similar and divergent viewpoints in accordance with the methodology [33]. Sub-categories were then sorted into categories through a process of abstraction and interpretation. The analysis was conducted by the authors (ER, ETP and HS), with the rest of the team having access to all data and participating in discussions. The codes and categories were compared to the data throughout the analysis to ensure that they reflected the material.

The analysis was conducted in Swedish. The sub-categories and main categories and the quotes selected to illustrate them, were translated into English by the authors.

##### Quantitative

Quantitative data from the pre- and post-surveys were imported from Microsoft Excel into Stata. Descriptive statistics of means were calculated from scores in the pre- and post-survey answers. Comparisons between pre- and post-survey answers were made using Wilcoxon rank sum test in Stata 15.1 (StataCorp, College Station, TX: Stata Corp LLC). A significance level of 0.05 was chosen.

### Ethical considerations

An application was sent to the Swedish Ethical Review Authority (no: 2021-01581). There were no ethical objections. The COREQ guidelines were adhered to. All informants received written and verbal information about the purpose of the study, emphasising the voluntary nature of participation and the right to decline or withdraw their given consent at any time, without having to give reasons. Written informed consent was given by each informant prior to the OMP workshop. Interviews were audiotaped and transcribed with consent, transcripts were pseudonymised. And access was restricted to the research team.

## Results

The analysis of the follow-up interviews regarding the informants’ experiences of OMP generated ten sub-categories, sorted into three main categories (Table 4). These are presented below, followed by the quantitative results from the surveys and a summary of the workshop evaluations.

**Table 4.** The main and Sub-categories generated through analysis of the interviews regarding the informants’ experiences of applying the OMP in their tutoring.

Main categories	1. The OMP – a flexible and easy-to-use model	2. The micro-skills serve as reminders of the key elements of clinical tutorship	3. Barriers and facilitators affecting the implementation of the OMP
Sub categories	1.1 A logical model facilitating clinical tutoring	2.1 A strength of the OMP is the five micro-skills combined in a clear structure	3.1 Barriers
	1.2 Useful regardless of students’ pre-existing levels of clinical experience and knowledge	2.2 Probing for underlying reasoning is useful for both students and clinical tutors	3.2 Facilitators
	1.3 Applicable in a variety of clinical situations	2.3 The OMP may foster more specific and frequent feedback and feedforward	
	1.4 Adaptable to the conditions of everyday clinical practice	2.4 Sometimes challenging to teach a rule of thumb, but useful for students	

**Table 5.** A comparison of tutors’ estimations of the frequency of using the micro-skills included in the OMP, as well as their competence in tutoring before and after the workshop.

Variable	Pre OMP workshop Mean (SD)	Post OMP workshop Mean (SD)	*p*-value
My attitude towards tutoring students is…	4.1 (0.7)	4.1 (0.7)	0.57
I feel competent as a tutor	3.9 (0.7)	4.2 (0.6)	0.05
I invite students to participate actively in clinical reasoning	4.3 (0.7)	4.4 (0.6)	0.75
I invite students to present suggestions on how to handle the patient case	4.0 (0.8)	4.2 (0.8)	0.07
I ask students to describe how they arrived at their suggestions	3.7 (0.8)	3.9 (0.9)	0.15
I try to assess whether students base their suggestions on adequate knowledge	4.1 (0.7)	4.0 (0.8)	0.70
I provide students with positive feedback	4.5 (0.6)	4.5 (0.6)	0.84
I provide students with feedback on what was *not* done well	3.8 (1.0)	4.2 (0.9)	0.03
I explain the reasoning behind my feedback	4.1 (0.7)	4.2 (0.9)	0.81
I give students specific suggestions on how to develop their clinical work further	3.9 (0.8)	4.1 (0.9)	0.14
I offer students a general ‘rule of thumb’ based on the present patient case	3.8 (0.8)	3.8 (1.0)	0.75

All items in the pre- and post-surveys use a five-point Likert scale. For variable 1, the response options were: 1= ‘very negative’, 5= ‘very positive’; for variable 2, 1= ‘disagree completely’, 5= ‘agree completely’; and for variables 3-11, 1= ‘not at all’, 5= ‘all the time’. The p-value was assumed to be significant at the 0.05 level.

### The OMP – a flexible and easy-to-use model

#### A logical model facilitating clinical tutoring

In general, the informants’ spontaneous evaluations of the OMP were positive; the model offered a logical structure which was easy to use and facilitated clinical tutoring. Some also commented on how the OMP increased their confidence as clinical tutors, since they believed they could offer students a better learning experience. Using the OMP also sparked reflection on tutorship in general:

But now you realise that even though you don’t always do 1, 2, 3, 4, 5, you still reflect on how it’s a bit more complex, that there is more you can actually, like, do in your tutorship to do it well, and yet it’s still not really more time-consuming. (Group3, nurse 2)

The OMP was also mentioned as a useful reminder of how tutoring is a task intertwined with – but also separate – from clinical work:

So you really get into all the medical stuff, but actually we’re supposed to tutor as well […] then I’m thinking this could help us find the focus in our tutorship amid everything else that we’re supposed to make time for. (Group 7, physician 4)

#### Useful regardless of students’ pre-existing levels of clinical experience and knowledge

Informants from both medical and nursing programmes used the OMP with students at all clinical levels. Some described they found it easier to apply with more clinically experienced students, while most argued that it did not matter since the OMP offers a set of generic steps which they could adapt according to the student’s performance:

If you feel like this [student] might be running his own race and so on, then maybe next time you put more effort into step two, like ‘Now you really have to tell me what you’ve been thinking and why’. (Group 8, nurse 2)

Another example of adaption was keeping the student’s level of knowledge in mind when choosing the rule of thumb taught.

Even though the workshop focused on applying the OMP at an undergraduate level, some informants also seamlessly extended its use to be part of their tool kit when mentoring at higher educational levels, for example, interns, residents or nurses in training.

#### Applicable in a variety of clinical situations

Informants reported having successfully applied the OMP in various clinical situations, including scheduled appointments, emergency visits and house calls. However, many physician informants preferred to use it in connection with student-booked patient encounters, one reason being that the time allotted was more generous. Discussions with students and feedback generally occurred without the patient present.

Some of the physician informants preferred the patient presenting with an undiagnosed complaint, as they found the OMP particularly useful for teaching clinical reasoning.

Nurse informants differed on whether the OMP was applicable in patient encounters with a pre-defined and limited cause. One example discussed was wound care. A few described this simply as a procedure to be taught, while others viewed it as an opportunity to apply the OMP for the student to reflect on and hopefully gain a deeper understanding of different types of wound characteristics, for example:

I believe you can expand your reasoning a lot in all sorts of situations, actually. Even when it comes to wounds and really think about sensation, smell and what it looked like previously. Could it be bacteria, virus, […] circulation? (Group 6, nurse 2)

#### Adaptable to the conditions of everyday clinical practice

Informants also found the model to be easily adaptable to the busy conditions of clinical practice. This was regarded as highly useful, since unexpected events – such as delays and unplanned interruptions, in addition to a high workload – were common.

Informants described adjusting the OMP on an ad hoc basis in two different ways: by either reducing the amount of time spent on each step, for example offering briefer feedback or ‘probing for underlying reasoning’ on a less detailed level, or by splitting up the structure and returning to omitted steps later in the day. For example:

Now [if] I’m seeing [several] patients with hypertension, and then it could be that for many of those it’s the same take-home messages. So then perhaps there is no need to go through it at each visit. (Group 8, nurse 1)

Some nurse informants claimed that it worked well to ‘save’ the OMP for scheduled reflection timed at the end of the day, instead of the intended in-between-patients tutoring session. Lack of time was mentioned as one reason for this.

### The micro-skills serve as reminders of the key elements of clinical tutorship

#### A strength of the OMP is the five micro-skills combined in a clear structure

Most informants recognised and had previously applied elements of the model, such as providing positive feedback, but not combined in a systematic structure. After having applied the OMP many considered the *combination* of the five micro-skills the major strength of the model. The combined micro-skills were described as effectively capturing the essential elements of tutoring by engaging the student and encouraging feedback.

It’s the model in its entirety that makes it good. (Group 2, physician 1)

With the OMP, tutoring was also described as a more structured activity, with the additional benefit of a reduced risk of ‘forgetting something’. Some also mentioned feeling more secure in their role as tutors with a structured toolkit instead of, for example, applying the ‘teach-them-everything-I-know strategy’:

Before this OMP model you didn’t have as much structure for how to deal with students, so you just tried to teach them what you knew yourself. (Group 3, nurse 1)

#### Probing for underlying reasoning is useful for both students and clinical tutors

Being reminded to encourage students to describe their reasoning (micro-skill 2) was considered by some informants to be the main benefit. Some described it as an advantage mainly for the students, since they, for example, got the chance to practise verbalising clinical reasoning,. Others emphasised how they felt supported in assessing the student as well. Assessing the student’s level of knowledge, particularly being able to identify unexpected gaps of knowledge, was seen by some as challenging but important:

But it’s the second one [‘probing for underlying reasoning’] where I see a great value, and which I personally haven’t been so good at before, to get a better understanding of how the student is reasoning. Because it tells you a lot about what you know and don’t know. (Group 6, physician 5)

Informants also described how, consequently, they now allowed the student more time to elaborate on their reasoning.

… now I’m much more careful about really trying to get [the student] to express it [their reasoning], which I think has also been very useful. So for me, that has been the greatest benefit and I think it is then also beneficial for the student. (Group 2, physician 1)

Some informants valued the reminder to ask open-ended reflective questions, as this stimulated dialogue with the student, which they considered to be an important component of tutorship.

…then you notice that the student finds it very useful to discuss with you. So I find that very positive. (Group 1, physician 2)

One of the nurse informants expressed a slightly deviating point of view on micro-skill 2, arguing that it was not applicable for nurses since it meant asking the student to account for clinical reasoning supporting diagnostic work, which in turn was associated with the physician’s role. This statement sparked a discussion in the group, as others disagreed and expressed that it was only a matter of training to be able to adjust the wording to fit the situation and the student, regardless of profession.

I believe you can practise developing your ability to ask reasoning questions. (Group 6, nurse 2)

Only a few of the informants commented on how allowing time for students to convey their reasoning may also facilitate giving specific feedback.

#### The OMP may foster more specific and frequent feedback and feedforward

Feedback and feedforward were widely discussed and most informants expressed views on this. Some described the feedback process as the most important aspect of tutoring.

It’s an important part because I also think that’s where you can really make a difference in your tutorship, that you really find the things that can be improved […] and dare to talk about them. (Group 5, physician 1)

In some of the interviews, there were discussions on how adhering to the OMP structure and giving students time to outline their reasoning could facilitate both positive feedback and feedforward, mainly by enabling it to be specific and timely.

Nevertheless, and regardless of the OMP, providing feedforward was still described by some as having negative connotations. However, there was also a comment on how the wording in the OMP guide (in Swedish) helped shift this perception.

But I think that step four expresses it well, that is: ‘Help the students to identify their shortcomings’. (Group 3, nurse 4)

Even so, many still described difficulties in verbalising feedforward. One reason mentioned was a perception of limited mandate, especially during short rotationsl. Another reason was unease about ‘critiquing’ the student:

But sometimes it feels a bit awkward to point out errors and shortcomings in a good way. You might feel that in general, well, you [the student] are doing very well so I really don’t want to say anything negative. (Group 7, physician 2)

However, routinely being reminded about providing feedforward by following the OMP steps was described as helpful.

Providing positive feedback was something most informants found relatively easy and familiar. Nonetheless, the OMP serving as a reminder not to overlook it was deemed useful by some.

…it says that I’m supposed to give positive feedback, so that works as a reminder for me. (Group 5, physician 2)

#### Sometimes challenging to teach a rule of thumb, but useful for students

Many informants saw teaching a rule of thumb in association with case presentations as new, since it was not previously done systematically. However, after applying the OMP, several recognised how a well-thought-out rule of thumb could be valuable for the students’ learning process. This was highlighted by some as a micro-skill they wanted to develop further:

…maybe you’ve talked about certain rules or that it’s important to consider this, but I haven’t thought about it in every conversation after every consultation, so I’ve been thinking that there is room for improvement there on my part. (Group 7, physician 4)

However, some considered this micro-skill challenging, and it would sometimes be omitted due to time constraints. Some tutors expressed that the rule of thumb taught should be evidence-based information, rather than practical or experience-based knowledge:

We use it when it’s possible and when there are rule of thumbs or other things to refer to like ‘Vårdhandboken’ [author’s translation: ‘guidebook of care’] […] or when it is something concrete that you have some evidence for and maybe can refer the student back to [the source]. But that might not always be possible. (Group 3, nurse 2)

### Barriers and facilitators affecting the implementation of the OMP

#### Barriers

While no barriers were specific to the OMP, the informants outlined several barriers to conduct clinical tutorship the way they wanted, which consequently also affected the implementation of the OMP. Taking time to get used to a new routine was mentioned along with recurring themes concerning lack of time and tutoring as a duty on top of the regular workload, with no time to think about implementing something new. One of the informants described how coming to work and facing a fully booked clinic, as well as a new student, resulted in returning to old habits, as there was no time to think or to prepare something new:

At that moment it didn’t even occur to me that this model existed. (Group 2, physician 2)

For some informants, several months passed between attending the OMP workshop and tutoring, which was also mentioned as a factor hindering implementation.

#### Facilitators

Informants felt the OMP’s micro-skills aligned with principles of clinical tutorship they had already learned, making the model easy to adopt:

It is almost the way you used to do things before you even understood that it was a model. (Group 8, nurse 4)

A positive workplace atmosphere towards clinical tutorship was also mentioned as a facilitator for implementation. This usually also meant more time for planning and for collegial discussionsin which they were able to share experiences of using the OMP. Some also found it valuable to inform students about the model at the start of the placement.

In addition, the laminated card summarising the micro-skills served as a useful ‘physical reminder’ for many of the tutors .:

I keep it on top of my drawers […] just to look at. You don’t need to bring it with you, it’s enough to just remind yourself ‘This is what I’m going to think about now’. (Group 2, physician 1)

## Quantitative data from the tutors’ pre- and post-surveys

The results showed a significant increase in the tutors’ self-rated competence in tutoring and in offering feedback on ‘what was not done so well’.

## Participant evaluation of the OMP workshop

The participants were asked to evaluate the three components of the workshop anonymously and individually, using a five-point Likert scale. The mean values were 4.44 for the Power Point presentation, 4.37 for the example video, and 4.25 for the role play. Most of the free text comments showed that the participants perceived the workshop as clear and instructive, with a good mix of theory and practice. Some would have liked more video examples, while others wanted more time for the role play. In general, the comments were positive and there were few concrete suggestions for improvement. Although some would have liked more instructions regarding the role play, which a few described as complicated.

## Discussion

The main findings from this study showed that a brief interprofessional workshop on the OMP was well received by clinical tutors in PHC. The OMP may support a more frequent, specific feedback process and improve their competence. Moreover, our qualitative data supported results from previous quantitative studies, showing that the OMP may foster clinical reasoning.

### How the OMP may foster clinical reasoning

After using the OMP, many informants highlighted the second micro-skill, ‘encouraging the student to describe their reasoning’, as having changed their tutorship. In ‘traditional’ tutoring, as described by Aagaard et al. [25] the focus is on ‘diagnosing the patient’, that is, the tutor functions as an expert consultant and focuses on asking questions about the patient’s problem. This, however, does not reveal the student’s thinking process. By contrast, the OMP has been described as a model which also ‘diagnoses the student’, meaning that as their reasoning is revealed, so too is their understanding of the case [25]. An overall positive impact on abilities for clinical reasoning for both undergraduate and post-graduate medical students has also been shown in a meta-analysis by Grunewald et al. [12]. Moreover, four out of twelve studies included in a systematic review by Gatewood et al. found that tutors’ assessments of students’ clinical reasoning skills were facilitated by the OMP [34]. Our qualitative data add to these previous findings by examining the tutors’ own perspectives: What do they describe doing differently, and how did that affect their tutoring? From our results, we deduce that the following may contribute to the OMP improving clinical reasoning:

First, for some of the informants, asking reflective questions was not part of their tutoring routine prior to the OMP. Instead, they were mostly used to presenting the student with suggestions on how to address the patient’s problem, in line with ‘traditional’ tutoring methods. However, by being reminded through the OMP to examine the student’s thinking process, many described having incorporated this strategy into their new routines. Second, the amount of time the informants allowed for the students to elaborate on their reasoning was something they estimated had increased (highlighted by some of the informants as the greatest change with the OMP), and the benefits gained from this seemed evident to the informants. For example, it was described as good practice for the student to reason out loud, which also facilitated the tutor’s assessment of the student. It is, however, noteworthy that the latter was not necessarily apparent to all informants, although this was a teaching point in the workshop. Finally, some informants described engaging in a dialogue with the student to a greater extent than previously, which is also a likely contributor to fostering clinical reasoning. The built-in structure of the model promoting collaborative dialogue and provision of feedback has also been highlighted by Pierce et al. as a distinctive feature of the OMP that contribute to its positive outcomes in clinical reasoning [11].

### The OMP may facilitate the feedback process

What Pierce et al. outlined in terms of the structure being helpful for the provision of feedback [11] was also supported by our study. In addition to allowing sufficient time for reasoning, the structure of the model was repeatedly mentioned as valuable for providing feedback.

Our quantitative data showed a significant increase in self-rated frequency of the use of feedforward, which was also supported by our qualitative results. Nevertheless, the informants also described difficulties with the feedback process, especially with feedforward. A previous study by Salerno et al. has shown that although the occurrence of feedforward increased significantly after OMP training, in general the frequency remained low [19]. Obstacles to providing feedforward in general are also reflected in previous research, which has indicated that – despite training – clinical tutors have difficulties transforming what they observe into constructive feedforward [30,35].

Moreover, sociocultural factors such as clinical workplace culture and relationships are known to influence the feedback process [30,36]. Although our informants perceived the OMP a facilitator, applying the model would not necessarily overcome sociocultural barriers. This was reflected in discussions among our informants where, for example, ‘not having a mandate to criticise’, ‘not wanting to upset the student’ and similar descriptions were mentioned as barriers. These phrasings used by the informants could be an indication of the emotional charge of the feedforward process. However, this may have been reduced for some of the tutors by our re-phrasing of the micro-skill ‘correct mistakes’ to ‘identify what could be done differently’. The latter has more neutral connotations, which may be helpful when starting to rethink the concept of feedforward.

Previous studies have also shown that the OMP may facilitate the feedback process. In Gatewood’s systematic review the authors concluded that the OMP improved teaching techniques and they also found a significant improvement in delivering feedback in five of the twelve studies included [34]. This is an important and valuable aspect of the OMP, since providing constructive feedback is still a domain of teaching where literature reports that clinical tutors often lack competence [30].

### The OMP improved self-rated competence

Our quantitative data showed a significant increase in tutors’ overall self-rated competence. Although not a main category in our qualitative data, some informants did mention feeling more competent as tutors after using the OMP. One reason given was being able to offer a better learning experience; another was having a consistent and reproducible structure to adhere to. A third reason may have been the fact that the model was coherent with their previous knowledge. Finally, by actively asking for underlying reasoning, instead of presuming knowledge, their confidence in making accurate assessments may have been supported.

### Implications for future interprofessional OMP workshops

Although their roles differ, there are many similarities between physicians and nurses working and tutoring in the primary care setting. Of importance for the possible implementation of OMP is that they are both responsible for their own patient encounters – that is, the basic framework for the OMP to be applied is in place. We found that an interprofessional OMP workshop is feasible, given that the material is adapted for both physicians and nurses. Both professions cited lack of time as a barrier, suggesting the OMP’s efficiency could have been stressed further. What a ‘rule of thumb’ could include was exemplified, but given the results it may have been helpful to further highlight the value of including experience-based knowledge.

Prior to the workshop sessions, our multidisciplinary research team worked on making the educational material accessible to both medical and nursing tutors. In general, according to our informants, this was achieved. Nonetheless, one group discussed whether the phrasing of one micro-skill emphasised diagnostics too narrowly. Although this was a deviating point of view among our informants, it is important to take into consideration – not least since a similar issue was highlighted by a previous study on OMP for nurses where a broader phrase ‘get the student to take a stand’ was chosen for the same reason [23]. This was considered in the Swedish translation but may need further refinement.

We were not able to control the distribution between the two professions in the various workshop groups due to logistical reasons. Although not an overt issue in our groups, achieving such balance in the future would help prevent power imbalances and encourage open discussion. Teachers representing both professions were present at the workshops for credibility and inclusivity which is also recommended for future implementation. Moreover, a follow-up session some weeks after the workshop would likely be beneficial to reinforce the OMP and address any questions and concerns, as. informants valued the opportunity to discuss their experiences in the follow-up interviews.

### Strengths and weaknesses

Given the conditions for conducting research in PHC settings, in terms of logistical (and economic) barriers which dominated the recruitment process for this study, we considered it a strength to succeed in achieving the goals set in the research plan, in which we estimated being able to conduct the workshop at 10 to 15 PHC centres and completing follow-up interviews until saturation.

Regarding the recruitment process, one weakness was that the contact persons at each PHC centre selected participants for the workshop and the follow-up interview. We were, however, able to check that our criteria for participation were fulfilled in the background data from the pre-survey. Strengths included a wide range of clinical and tutoring experience, balanced representation of physicians and nurses from both genders.

Data collection was affected by busy schedules, which resulted in missing data for the pre-survey as some of the workshop participants arrived late. A larger number of informants were planned to participate in the interviews, but changes often occurred last minute, which unfortunately meant that we ended up with fewer informants. Moreover, the response rate for the post-survey was lower than expected, likely due to it not being a priority for the participants during their busy clinic workday. The student survey had an even lower response rate making the data unusable for analysis. There is a known to be survey fatigue among students, which was likely the main cause of this.

Finally, by relying on experiential learning theories from medical education, the authors may have overlooked valuable perspectives from other fields. However, this focus ensures the discussion remains directly relevant and applicable to both students and tutors in the medical context.

## Conclusion

Our study adds a qualitative perspective to previous research showing that the OMP benefits students’ clinical reasoning, the feedback process and tutor competence. Moreover, we found that an interprofessional OMP workshop was well-received among tutors in PHC, and considering suggestions for future implementation, scaling up is likely to be feasible. In conclusion, we believe that introducing the OMP on a larger scale to tutors may improve clinical training for students, particularly by fostering their ability for clinical reasoning through reflective questions being asked rather than answers being given.
